# Complete Genome Sequence of Klebsiella pneumoniae Siphophage Sanco

**DOI:** 10.1128/MRA.01252-19

**Published:** 2019-10-31

**Authors:** Ryan W. Richardson, Lauren Lessor, Chandler O’Leary, Jason Gill, Mei Liu

**Affiliations:** a Center for Phage Technology, Texas A&M University, College Station, Texas, USA; DOE Joint Genome Institute

## Abstract

Klebsiella pneumoniae is an opportunistic pathogen that is the cause of several hospital-acquired infections. Bacteriophages that target this bacterium could be used therapeutically as novel antimicrobial agents. Here, we present the complete genome sequence of the T1-like K. pneumoniae phage Sanco.

## ANNOUNCEMENT

Klebsiella pneumoniae, especially with the continued emergence of multidrug-resistant strains, has become a significant public health threat in hospital settings (1–3). Characterization of K. pneumoniae phages may prove useful in developing new treatments for infections caused by this bacterium.

Phage Sanco was isolated in 2013 from a wastewater treatment plant in College Station, TX, against a deidentified K. pneumoniae clinical isolate. K. pneumoniae was cultured on tryptic soy broth or agar (Difco) at 37°C with aeration. Phage were cultured and propagated by the soft-agar overlay method (4). It was identified as a siphophage using negative-staining transmission electron microscopy, performed at the Texas A&M University Microscopy and Imaging Center as described previously (5). Phage genomic DNA was prepared using a modified Promega Wizard DNA cleanup kit protocol (5). Pooled indexed DNA libraries were prepared using the Illumina TruSeq Nano LT kit, and the sequence was obtained with the Illumina MiSeq platform using the MiSeq v2 500-cycle reagent kit following the manufacturer’s instructions, producing 1,296,046 paired-end reads for the index containing the phage Sanco genome. FastQC v0.11.5 (https://www.bioinformatics.babraham.ac.uk/projects/fastqc/) was used to quality control reads. The reads were trimmed with FastX Toolkit v0.0.14 (http://hannonlab.cshl.edu/fastx_toolkit/download.html) before being assembled using SPAdes v3.5.0 (6). Contig completion was confirmed by PCR using primers 5′-CCGGTTTGTCGATATCATCC-3′ and 5′-ACGGAGGTGTTTTCAATCCA-3′ facing off the ends of the assembled contig and Sanger sequencing of the resulting product, with the contig sequence manually corrected to match the resulting Sanger sequencing read. GLIMMER v3.0 (7) and MetaGeneAnnotator 1.0 (8) were used to predict protein coding genes with manual verification, and tRNA genes were predicted with ARAGORN 2.36 (9). Rho-independent termination sites were identified via TransTermHP (http://transterm.cbcb.umd.edu/). Sequence similarity searches were done by using BLASTp v2.2.28 (10) against the NCBI nonredundant (nr) and UniProt Swiss-Prot (11) and TrEMBL databases. InterProScan 5.15-54.0 (12), LipoP (13), and TMHMM v2.0 (14) were used to predict protein function. HHpred with ummiclust30_2018_08 for multiple sequence alignment (MSA) generation and PDB_mmCIF70 for modeling in the HHsuite v3.0 release were also used for functional prediction (15). All analyses were conducted using default settings via the Center for Phage Technology (CPT) Galaxy (16) and WebApollo (17) interfaces (https://cpt.tamu.edu/galaxy-pub).

Sanco was assembled at 28-fold coverage into a complete contig of 48,790 bp. The GC content of the genome is 51%, which is lower than that of the host (57%) (18). Determined by BLASTn against the NCBI nucleotide (nt) database, Sanco shares greater than 83% overall nucleotide identity (E value = 0) with a group of characterized T1-like *Klebsiella* phages, including Sushi (GenBank accession no. KT001920) (19), Skenny (MK931444) (20), Sweeny (MK931443) (21), and Shelby (MK931445) (22). The Sanco genome was opened to be syntenic with those of phage T1 (NC_005833) and phage TLS (NC_009540). Sanco proteins sharing homology (determined by BLASTp search against the NCBI nr database at an E value cutoff of 10^−3^) with T1 proteins include those involved in phage morphogenesis and DNA replication. A full lysis cassette was identified in the Sanco genome, and it included a holin, signal-arrest-release (SAR) endolysin, and unimolecular spanin. The endolysin is predicted to have a glycosidase activity.

### Data availability.

The genome sequence of phage Sanco was submitted to GenBank under accession no. MK618657. The associated BioProject, SRA, and BioSample accession numbers are PRJNA222858, SRR8772108, SAMN11236500, respectively.
